# Residual Deformity of the Trochlea After Non-displaced Supracondylar Fracture in a Child: A Case Report

**DOI:** 10.7759/cureus.54734

**Published:** 2024-02-22

**Authors:** Hideaki Ishii, Takanori Shintaku, Shu Yoshizawa, Takahiro Maeda, Hiroyasu Ikegami

**Affiliations:** 1 Department of Orthopaedic Surgery, Toho University Ohashi Medical Center, Tokyo, JPN

**Keywords:** conservative treatment, distal humerus fracture, supracondylar fracture, fishtail deformity, elbow

## Abstract

Residual deformity of the trochlea after fractures of the distal end of the humerus in children is well known and is referred to as fishtail deformity. Despite numerous reports on this entity, the reason for various types of fractures with the same results remains unknown. Fishtail deformities after non-displaced supracondylar fractures are very rare. A 7-year-old boy with a non-displaced supracondylar fracture was treated conservatively. Three years later, the patient returned to our hospital complaining of mild elbow pain. Radiography revealed a fishtail deformity of the trochlea due to the premature fusion of the epiphysis. At the latest follow-up at the age of 17 years, only a marginal limitation at the excursion of the elbow was observed, and no additional treatment was needed. Fishtail deformities can occur even after a non-displaced supracondylar fracture. Long-term follow-ups are required in children with distal humeral fractures.

## Introduction

Supracondylar fracture of the humerus is the most common fracture, accounting for approximately 60% of all elbow fractures in children [1]. Many types of sequelae, such as neurovascular impairment, angular deformities, premature growth arrest, avascular necrosis, malunion of the distal humerus, and stiffness of the elbow, can occur after supracondylar fracture, regardless of the amount of displacement or treatment methods [2]. Fishtail deformity of the distal humerus is a rare but non-negligible delayed complication of supracondylar fractures in children [3]. Although very rare, fishtail deformities can occur after supracondylar fractures without displacement, and has been reported in only a few cases [4,5]. Symptoms often do not appear for the first few years following discovery in most cases. A fishtail deformity might predispose patients to the development of osteoarthritis and result in functional disability later in life [6]. We report a case of fishtail deformity after a non-displaced supracondylar fracture with a follow-up period of more than 10 years.

## Case presentation

**Figure 1:**
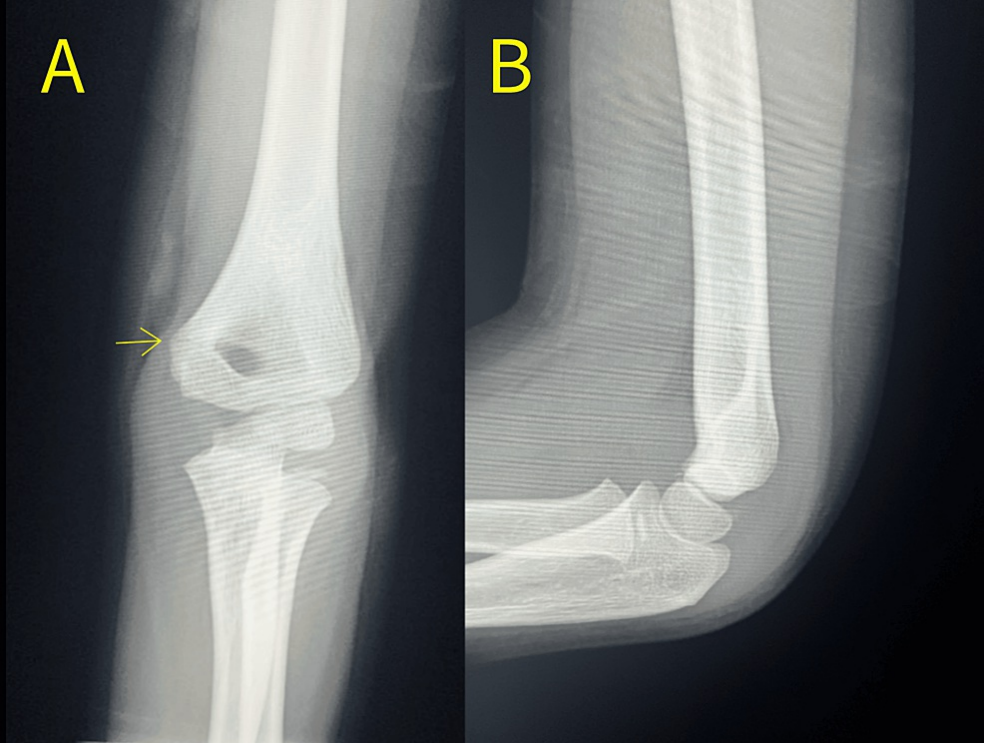
X-ray of anteroposterior (A) and lateral (B) views of the elbow joint on the day of initial injury. Anteroposterior view (A) shows a supracondylar fracture of the distal humerus without displacement.

A right-hand-dominant boy aged seven years and eight months fell from a horizontal bar about 1.5 meters high with his left elbow extended and sustained a non-displaced supracondylar fracture (Gartland criteria type I) of the distal humerus (Figure 1). A physical examination revealed slight swelling and pain in the elbow, but no signs of nerve palsy or circulatory disturbance. Thus, no additional studies other than radiography were performed during the first visit.

**Figure 2:**
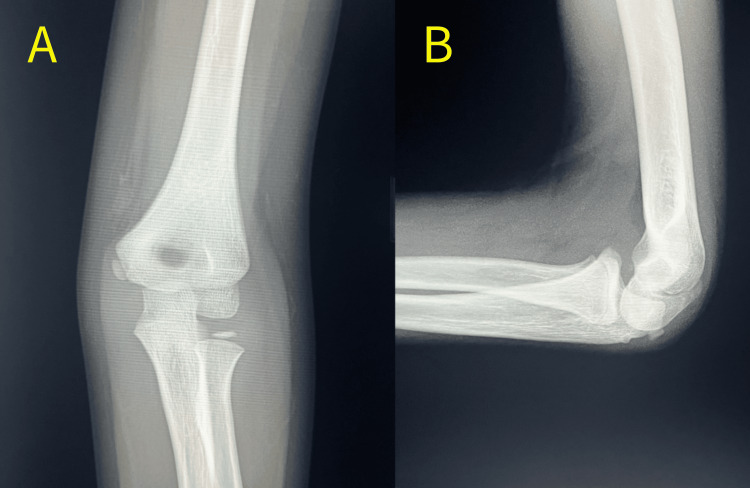
X-ray of anteroposterior (A) and lateral (B) views of the elbow joint six weeks after the initial injury. Bone healing without any deformation was obtained.

He was treated conservatively with 80° of elbow flexion and neutral forearm rotation using a long-arm splint for three days. At the second visit, normal motor, sensory, and circulatory functions were confirmed, which were then changed to a long-arm cast with the elbow at 90° flexion. The cast was removed four weeks after the injury, and active range of motion (ROM) exercises for the elbow were permitted. At the six-week follow-up, radiography revealed synostosis of the fracture site without displacement or deformity (Figure 2). The patient complained of no pain, and a completely normal ROM of the elbow (5° of extension, 130° of flexion, 90° of pronation and supination), equivalent to that of the unaffected side, was achieved.

**Figure 3:**
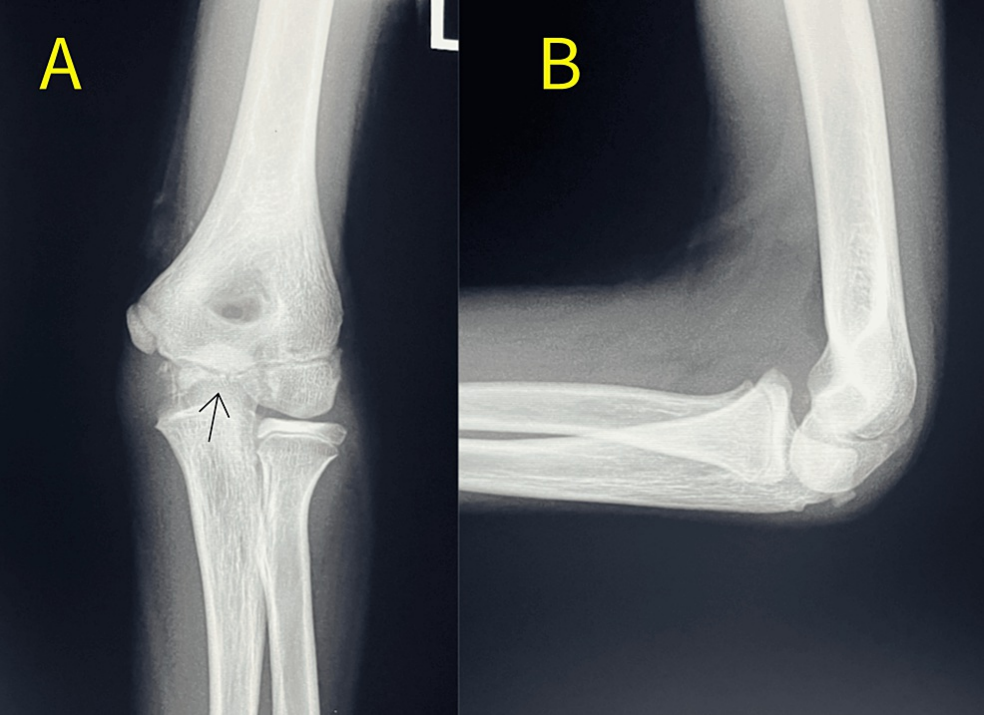
X-ray of anteroposterior (A) and lateral (B) views of the elbow joint three years after initial injury. The distal end of the humerus is not completely ossified. However, a deformity of the articular surface at the trochlea is suspected.

**Figure 4:**
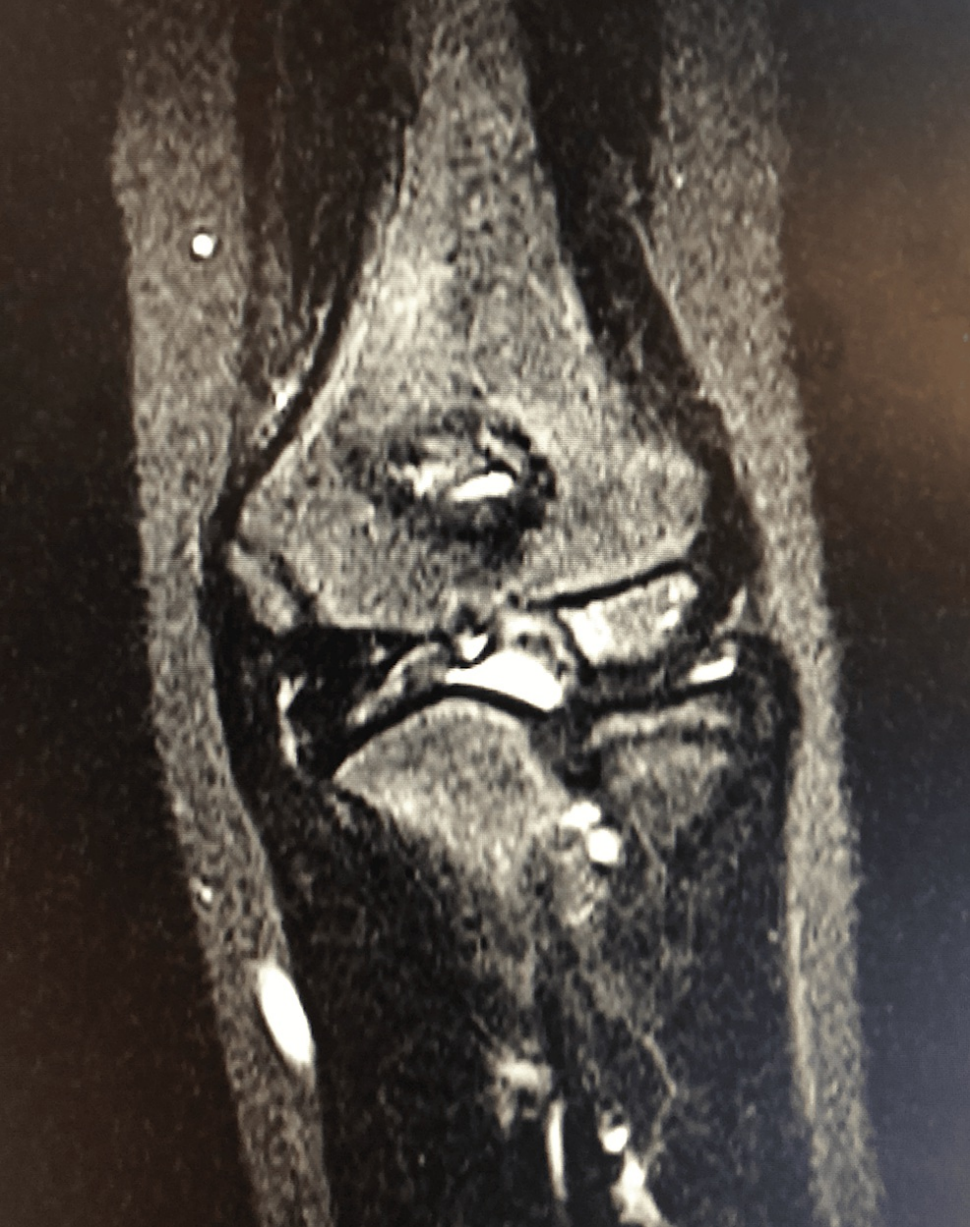
STIR coronal section MRI of the elbow joint after the second injury. Hydrarthrosis and deformity of the lateral part of the trochlea are apparent. STIR: Short tau inversion recovery.

Three years after the fracture, he revisited our hospital complaining of pain in the left elbow while jumping over a vaulting horse. Radiography of the elbow revealed an irregular articular surface of the capitellum and trochlear deformity (Figure 3). A fishtail deformity is classified as type A according to Wilkins et al., which involves the lateral portion of the medial Christa apex of the trochlea [7]. MRI of the elbow performed one week after the second injury showed a bone bruise and a partial chondral defect of the capitellum, hydrarthrosis, and deformity of the lateral part of the trochlea (Figure 4).

**Figure 5:**
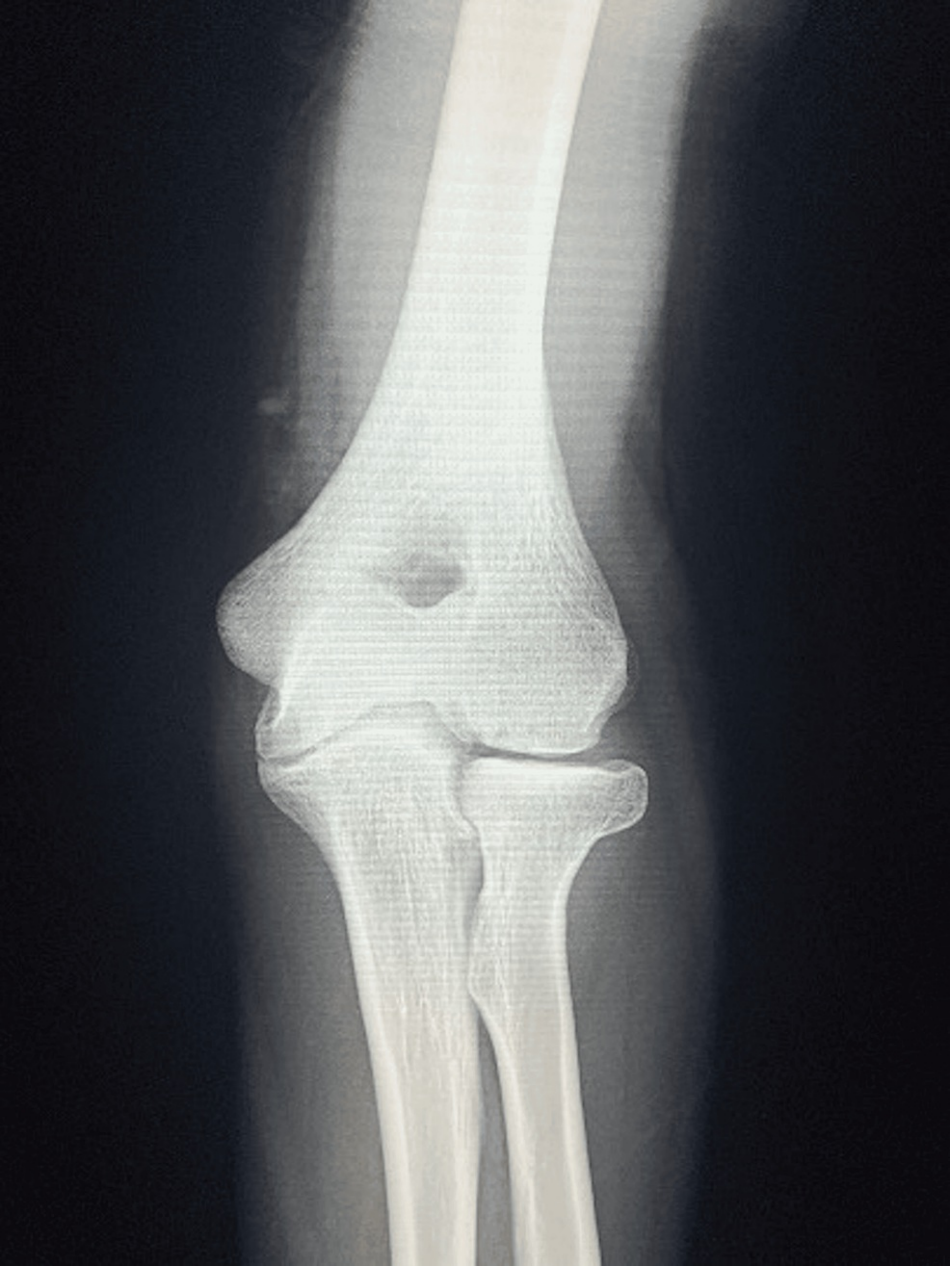
X-ray of the elbow joint 10 years after initial injury. Fishtail deformity due to growth impediment of the lateral part of the trochlea and compensatory hypertrophy of the radial head is evident.

It was a consolation that the congruity of the ulnohumeral joint was preserved; thus, the patient underwent conservative treatment again and was kept under observation. The carrying angle was 18° on the unaffected side while it was 12° on the affected side in radiographic measurements and a fishtail deformity was evident four years after the initial injury, there was no complaint of persistent pain nor neurological issues at the elbow. Annual follow-up is still ongoing at the age of 17 years, and partial deformity of the trochlea has passed without progression (Figure 5), with mild limitation of ROM of the elbow (130° in flexion and -20° in extension), but no limitation in range of forearm (90° in pronation and 90° in supination) nor significant change of carrying angle.

## Discussion

We report a case of fishtail deformity of the distal humerus that became evident three years after a non-displaced supracondylar fracture. Fishtail deformity of the distal humerus is a rare and delayed complication of distal humeral fractures in children [3]. In 1948, McDonnell and Wilson first reported avascular necrosis in the distal humeral epiphysis as a sequence of distal humeral fractures, which included four cases of supracondylar fractures [8]. Wilson first used the term "fishtail deformity" in 1955 to describe the shape of the deformed distal humerus caused by an undeveloped lateral trochlea after a lateral condylar fracture [6]. Since then, several cases of fishtail deformity have been reported in children. As time progressed, it became clear that fishtail deformities can occur after almost any kind of distal humeral fracture, including lateral condyle fractures, medial condyle fractures, physeal separations, and supracondylar fractures [2,3]. Among these fractures, most fishtail deformities have been reported to occur because of displacement or surgical invasion. To the best of our knowledge, reports of fishtail deformity as a sequela of non-displaced supracondylar fractures treated conservatively without reduction are extremely rare, with only few cases reported in the English literature prior to our case [4,9].

Vascular insults and idiopathic growth disturbances are two major theories concerning the etiology of fishtail deformity [3,9]. Although no conclusion has been reached as to which of these two theories is veracious, the vascular theory, which has been favored in recent reviews, is supported because of its anatomical features [9]. Haraldsson investigated the blood flow at the distal end of the humerus in children and reported that the glenoid region has lateral vessels that nourish the apex to the outside of the glenoid and medial vessels that nourish the inside of the glenoid, which do not anastomose with each other [10]. They discovered that the trochlear ossification nucleus was supplied by a terminal vessel. Yang et al. studied the microcirculation of the distal humeral epiphyseal cartilage and demonstrated longitudinal vascularity in the epiphyseal cartilage, with no communication between the capitellum and trochlea [11]. They advocated that disruption of either the longitudinal intraosseous vasculature or the vascular arch in more than two places may lead to selective avascular necrosis of the epiphyseal cartilage between the capitellum and trochlea. 

Wilkins et al. proposed two patterns of avascular necrosis in the distal humerus [7]. Type A involves the lateral part of the medial Christa apex of the trochlea, which is considered a classical fishtail deformity, mainly observed after supracondylar and lateral condylar fractures. Type B involves the entire trochlea and often some part of the medial metaphysis in older children or after medial condylar fractures. Our case resulted in a type A fishtail deformity according to the classification by Wilkins et al. [7]. Etier et al. hypothesized that the etiology of vascular disruption in non-displaced supracondylar humeral fractures involves the tamponade effect [9]. Non-displaced fractures result in a fractured hematoma contained in an intact capsule, with the potential to increase pressure and lead to occlusion of the lateral, intra-articular vessels, which might result in a type A fishtail deformity. This theory may explain our case. However, the actual mechanism of this deformation is yet to be elucidated.

Recently, the term fishtail deformity has no longer been very rare for many pediatric orthopedists after distal humeral fractures; however, its importance in clinical practice is not yet widely recognized. This is due to the rarity and absence of symptoms during childhood. It should be noted that the degree of deformation can increase with growth, and problems, such as cracking, pain, or loss of mobility can occur when the humeroulnar joint is affected. Surgical treatment may be an option for patients with significant functional impairment.

As fishtail deformities can cause problems later in life, the longest possible follow-up until bone maturation is desirable. Although it is very difficult to follow up on all cases over a long period after a supracondylar fracture of the elbow, we have a responsibility to inform parents to return for follow-up if the patient complains of any kind of discomfort in the elbow in the future. It must be noted that fishtail deformities can occur after almost any type of distal humeral fracture, including non-displaced supracondylar fractures. The deformity becomes more pronounced with bone maturity, and long-term follow-up is required for distal humeral fractures in children.

## Conclusions

Fishtail deformities can occur after almost any type of distal humeral fracture, including non-displaced supracondylar fractures. The deformity becomes more pronounced with bone maturity, and long-term follow-up is required for distal humeral fractures in children.
